# From glucose lowering agents to disease/diabetes modifying drugs: a “SIMPLE” approach for the treatment of type 2 diabetes

**DOI:** 10.1186/s12933-021-01281-y

**Published:** 2021-04-28

**Authors:** Ofri Mosenzon, Stefano Del Prato, Meir Schechter, Lawrence A. Leiter, Antonio Ceriello, Ralph A. DeFronzo, Itamar Raz

**Affiliations:** 1 The Diabetes Unit, Department of Endocrinology and Metabolism, Hadassah Medical Center, P.O. Box 12000, 9112001 Jerusalem, Israel; 2 Faculty of Medicine, Hebrew University of Jerusalem, Jerusalem, Israel; 3 Department of Clinical and Experimental Medicine, Section of Diabetes, Nuovo Ospedale Santa Chiara, University of Pisa, Pisa, Italy; 4 Li Ka Shing Knowledge Institute, St. Michael’s Hospital, University of Toronto, Toronto, Canada; 5 IRCCS MultiMedica, Sesto San Giovanni, Milan, Italy; 6 University of Texas Health Science Center at Houston, Houston, TX USA

**Keywords:** Diabetes/Disease Modifying Drugs (DMDs), Type 2 Diabetes, Clinical approach

## Abstract

During the last decade we experienced a surge in the number of glucose lowering agents that can be used to treat patients with type 2 diabetes. Especially important are the discoveries that sodium glucose co-transporter type 2 inhibitors (SGLT2i) and glucagon-like peptide-1 receptor agonists (GLP-1 RA) improve patients’ cardiovascular and renal outcomes. Accordingly, various medical associations have updated their guidelines for the treatment of diabetes in this new era. Though not agreeing on every issue, these position-statements generally share a detailed and often complex workflow that may be too complicated for the busy and overworked primary care setting, where the majority of patients with type 2 diabetes are managed in many countries. Other guidelines, generally those from the cardiology associations focus primarily on the population of patients with high risk for or pre-existing cardiovascular disease, which represent only the minority of patients with type 2 diabetes. We believe that we should re-define SGLT2i and GLP-1 RA as diabetes/disease modifying drugs (DMDs) given the recent evidence of their cardiovascular and renal benefits. Based on this definition we have designed a SIMPLE approach in order to assist primary care teams in selecting the most appropriate therapy for their patients. We believe that most subjects newly diagnosed with type 2 diabetes should initiate early combination therapy with metformin and a prognosis changing DMD. The decision whether to use GLP-1 RA or SGLT2i should be made based on specific patient’s risk factors and preferences. Importantly, DMDs are known to have a generally safe side-effect profile, with lower risk for hypoglycemia and weight gain, further promoting their wider usage. Early combination therapy with DMDs may improve the multiple pathophysiological abnormalities responsible for type 2 diabetes and its complications, thus resulting in the greatest long term benefits.

## Simple, Initial combination therapy, Multiple risk reduction, Primary care team, Life changing/Prevention, re-Evaluation (Box 1)


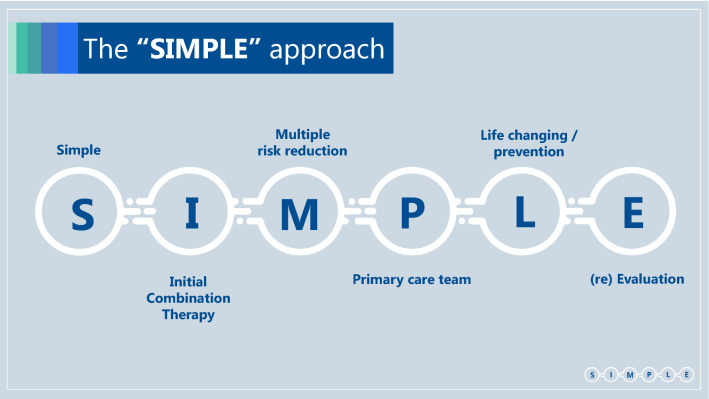


### Simple

The new millennium has brought a new era to the treatment of type 2 diabetes, with a surge in the number of available therapies [1]. Concomitantly, regulators have required pharmaceutical companies to demonstrate the cardiovascular (CV) safety of these new glucose lowering agents (GLAs), leading to a large number of completed cardiovascular outcomes trials (CVOTs) [2] which have generated vast amounts of new information. The position statements of the ADA/EASD [3, 4] ECS/EASD[5], AACE/ACE[6] and many others [7] are generally quite complex and therefore applying them to a specific patient can be challenging and time consuming. Since the primary care teams manage most patients with type 2 diabetes and are very often pressed for time, this complexity is a major barrier limiting the implementation of these guidelines in common practice [8].

**Fig. 1:**
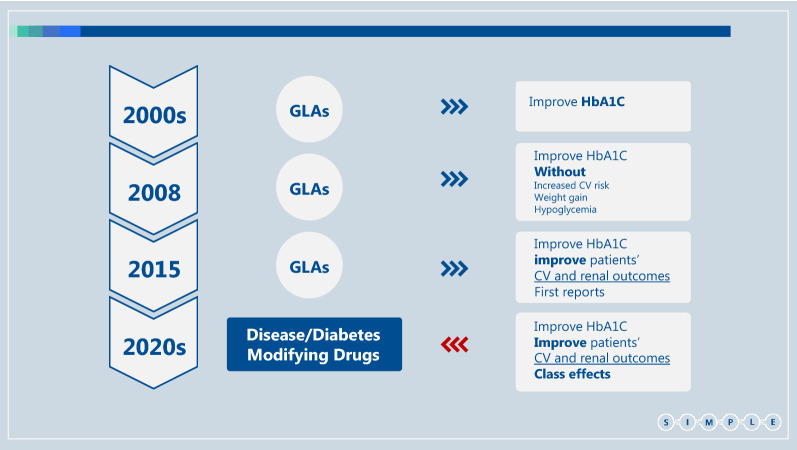
Changes in the treatment of patients with type 2 diabetes over the last two decades. *GLA* Glucose Lowering Agents, *CV* Cardiovascular, *FDA* Food and Drugs Administration, *DPP4i* Dipeptidyl peptidase-4 inhibitors, *SGLT2i* Sodium glucose co-transporter type 2 inhibitor, *GLP-1 RA* Glucagon-like peptide-1 receptor agonists, *DMD* Disease/Diabetes modifying drug, *MACE* major adverse cardiovascular events. Until the early 2000s treatment of type 2 diabetes focused on glucose control. The main GLAs in use were insulin, metformin and sulfonylureas. In 2008, the FDA started to require pharmaceutical companies to confirm the CV safety of their newly developed GLAs, compared with placebo or other GLA, in patients with high CV risk [82]. Members of the DPP4i and thiazolidinediones classes were found to have CV non-inferiority, without CV superiority, in MACE-based outcomes [55, 83–86]. Starting in 2015, several members of the SGLT2i and GLP-1 RA classes were found to exert cardiorenal protection [10–13, 15–17, 48, 56], defining them as disease/diabetes modifying drug (DMDs)

Based on recent literature we believe that this intricacy can be minimized. Recent advances have led to definition of a new family of medications which we term “diabetes/disease modifying drugs” (DMDs). DMDs are GLAs that have CV [including atherosclerotic cardiovascular disease (ASCVD) and/or heart failure (HF)] and/or renal protective effects demonstrated in a large multicenter, multinational, randomized, placebo-controlled clinical trial (Fig. 1). According to this definition most glucagon-like peptide-1 receptor agonists (GLP-1 RA)s and sodium glucose co-transporter type 2 inhibitors (SGLT2i) are DMDs [9–17]. The benefit of DMDs extend far beyond their glucose control effect, and also include weight reduction. DMDs have a blood pressure lowering effect; SGLT2i through their function in the kidney and GLP-1 RAs probably through reduction in body weight. GLP-1 RAs may also improve liver function tests. Most importantly, DMDs exert robust beneficial effects on the kidney and on the heart as a result of these and other yet unknown mechanisms [18–24]. Most patients with type 2 diabetes across different sub-populations may benefit from DMDs; we should therefore strive to treat most type 2 diabetes patients with DMDs, even those without specific risk factors. This definition makes treatment approaches simpler and therefore more useful in the primary care setting. Here we propose a SIMPLE approach to the treatment of type 2 diabetes that is based on the recent development and characterization of DMDs.

### Initial combination therapy

Many older GLAs have been associated with significant risk for hypoglycemia and weight gain, leading to a cautious step-wise treatment strategy often described as “treat to failure” i.e. treatment initiation with a single agent and adding a second one only after the loss of plasma glucose control [25]. This approach was supported by large trials (ACCORD, ADVANCE and VADT) that did not find significant advantages for intensive glucose control (e.g. HbA1c below 6.0% or 6.5%) in patients with relatively long diabetes duration (median of 8.0–11.5 years), on CV outcomes; this paradigm was especially relevant in patients with previous CVD [26–28]. However, such practice may result in long periods in which patient’s blood glucose levels are not well controlled, increasing the risk for future complications [29]. Newer DMDs, with beneficial effects on weight and lower occurrence of hypoglycemia, provide us with a valuable opportunity to harmonize the gluco- and cardio-centric approaches in diabetes management. We therefore argue for an “early combination” approach, starting with metformin and a DMD [30–32]. Importantly, this “early combination therapy” practice is well supported by up-to-date literature. DeFronzo and Abdul-Ghani demonstrated the beneficial effect of early combination therapy on markers of preservation of beta-cell function [33]. Furthermore, large retrospective cohorts [34, 35], a meta-analysis of 15 randomized controlled trials (RCT)s[36] as well as a recent large long-term RCT (the VERIFY trial)[31, 37] have all shown that early combination therapy leads to earlier, better and long-standing glucose control. The results of the NIH-supported GRADE study, an ongoing trial comparing different early combination therapies, will hopefully provide more information regarding the right composition for improved glucose control. However, the tested drugs include GLP-1 RA but not SGLT2i, and participation was not restricted to patients with newly diagnosed type 2 diabetes, resulting in an average baseline diabetes duration of approximately 4 years. Notably, RCTs data is lacking regarding the effect of early combination therapy on hard CV and kidney outcomes.

Thanks to the reduced risk of hypoglycemia with many agents including metformin, thiazolidinediones (TZDs), dipeptidyl peptidase 4 inhibitors (DPP4i), GLP-1 RA and SGLT2i, we advocate aiming for tighter glucose control. We acknowledge that the specific target HbA1c for most patients is still controversial, however until further research is conducted with safer DMDs, we generally suggest an HbA1c target of 6·5% or lower. Of course, this should be done cautiously and according to patient’s characteristics—excluding patients with history of severe hypoglycemia and those coping with comorbid conditions associated with frailty and/or limited life expectancy. Aiming for tighter glucose levels will enable longer intervals between visits, thereby reducing the risk for clinical inertia. Importantly, this target is supported by recent data indicating that more physiological plasma glucose levels at early stages limit glucose toxicity and intervene with other disease mechanisms—possibly halting its progression [33, 38, 39]. For example, in subjects with pre-diabetes, reversion to normal glucose values, even temporarily, was associated with a marked delay in the incidence of diabetes, as well as improved beta-cell function and increased insulin sensitivity [38]. In patients with type 2 diabetes, longer periods of poor glycemic control was correlated with lower likelihood of attaining glycemic control once treatment was intensified [35]. Furthermore, early tight glucose control may be associated with lower rates of type 2 diabetes complications [30, 40]. For instance, HbA1c < 6.5% during the first year from type 2 diabetes diagnosis, was recently associated with reduced risk for both microvascular and macrovascular complications, assessed during 10-years follow-up period [29]. Although the participants in these cohorts were mostly treated with older GLAs, similar studies with newer DMDs might yield similar or better results with lower concerns for side effects. It is time for earlier combination therapy, more physiological plasma glucose targets, and a stronger call to avoid the risk of treatment inertia.

We are aware of the recent controversy regarding the place of metformin in this new era of DMDs. Certainly, the great efficacy and safety of metformin over the years cannot and should not be overlooked. Relevantly, the UKPDS trial demonstrated the CV efficacy of metformin, compared to the conventional therapy at the time, in a small number of patients [41, 42]. While in patients already treated with sulfonylureas, the addition of metformin was associated with a mild increase in diabetes-related deaths [41]; however, in our approach we limit the use of sulfonylureas. It is important to note that all recent CVOTs with DMDs included about 80% of patients already treated with metformin.

Finally, we believe that there is a population of patients who require insulin therapy at diagnosis—specifically, those with symptomatic hyperglycemia and/or HbA1c > 9.0% [43]. We are concerned that due to the wide spectrum of other therapeutic options, insulin use may be deferred even for those who can benefit from it at an early stage [44]. Importantly, recent data indicates that early combination of insulin and GLP-1 RA or other GLAs lead to better glycemic control [45, 46].

### Multiple risk reduction

**Fig. 2:**
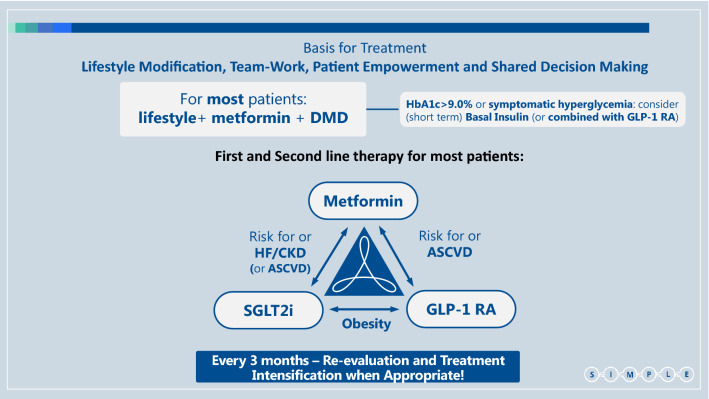
The choice of glucose lowering agents. Established ASCVD: Prior proven coronary artery disease, cerebrovascular disease or peripheral arterial disease**.** Risk factors for ASCVD: Older age (male > 50, female > 55) and one or more risk factor(s) for cardiovascular disease - HTN, hyperlipidemia, (current) smoker. HF: Prior clinical or imaging diagnosis of HFrEF. Risk factors for HF: According to TIMI risk-score [47]**.** CKD: eGFR < 90 ml/min/1.73 m^2^ and/or UACR ≥ 30 mg/g. Obesity: BMI > 30 kg/m^2^. *ASCVD* Atherosclerotic cardiovascular disease, *HTN* Hypertension, *HF* Heart failure, *HFpEF* Heart failure with preserved ejection fraction, *HFrEF* Heart failure with reduced ejection fraction, *CKD* Chronic kidney disease, *eGFR* estimated glomerular filtration rate, *UACR* Urinary albumin to creatinine ratio, *BMI* Body mass index, *SGLT2i* Sodium glucose co-transporter type 2 inhibitor, *GLP-1 RA* Glucagon-like peptide-1 receptor agonists, *DMD* Disease/Diabetes modifying drug (either SGLT2i or GLP-1 RA)

We propose a simple workflow to assist physicians and specifically primary care teams, in the process of drug selection (Fig. 2). It includes suggestions for early combination therapy, not only according to patient HbA1c but mainly according to his/her risk factors and co-morbidities.

We suggest a combination of SGLT2i with metformin in patients at increased risk for chronic kidney disease (estimated glomerular filtration rate (eGFR) < 90 ml/min/1·73m^2^ and/or urinary albumin to creatinine ratio (UACR) ≥ 30 mg/g) or for heart failure [according to TIMI-Hadassah risk score[47]] and possibly also in patients with established cardiovascular disease (eCVD)—although the effect of SGLT2i in the reduction of atherosclerotic events in patients with eCVD may be less consistent than previously thought, with the recent reporting of the non-inferiority results of the VERTIS-CV trial [48]. For patients at high risk for/or with ASCVD, we suggest a combination of metformin with GLP-1 RA (Fig. 2). A combination of GLP-1 RA and SGLT2i should be considered in patients where obesity is the main concern, although data on this is limited [49–52]. Of note, oral semaglutide is the first oral GLP-1 RA that was recently approved by the Food and Drug Administration (FDA) [53]. This will hopefully lead to earlier and wider use of GLP-1 RA [54], with their proven advantages. Often a patient will eventually need the combination of all three groups of drugs: metformin + SGLT2i + GLP-1 RA.

In the process of selecting the right GLA\DMD within the same group of agents, we should keep in mind the between-drug variability, the specific populations in which the drug was investigated, and results obtained for each drug. Table 1 summarizes the main safety and efficacy findings obtained for each drug.

**Table 1:** Classes of glucose lowering agents: main findings of CVOTs and other important trials

Group of drugs	Specific brands	Dosing	Comments	CVOTs / CROTs
SGLT2 inhibitors	Canagliflozin	100, 300 mg QD	Proven reduction in 3-point MACE, hHF, all-cause mortality and ”hard” renal outcomes in population of patients with/or risk factors for/ or previous ASCVD, as well as in population of patients with proteinuric diabetic nephropathy. Increased risk for fractures and amputations in one outcome trial (CANVAS) but not in the other (CREDENCE)	CANVAS Program [13, 87] CREDENCE [69]
	Dapagliflozin	5, 10 mg QD	Proven reduction in hHF/CVD and ”hard” renal outcomes in population of patients with risk factors for/or previous ASCVD. Proven reduction in CV death and hHF in populations of patients with HFrEF with or without diabetes. Proven reduction in a composite of clinically important kidney outcomes and renal or CV death, in patients with CKD with or without diabetes	DECLARE-TIMI 58 [16, 88] DAPA-HF [89] DAPA-CKD [70]
	Empagliflozin	10, 25 mg QD	Proven reduction in 3-point MACE, CV death, hHF, all-cause mortality and ”hard” renal outcomes in populations of patients with previous ASCVD. Proven reduction in CV death and hHF in populations of patients with HFrEF with or without diabetes	EMPA-REG OUTCOME [10, 56] EMPEROR-Reduced [90]
	Ertugliflozin	5, 15 mg QD	CVOT in population of patients with ASCVD reported CV safety both regarding 3-point MACE and CVD/hHF. Lower rate of hHF, and a trend towards improved renal outcome	VERTIS-CV [48]
SGLT2 & SGLT1 inhibitor	Sotagliflozin	200, 400 mg QD	Reduction in CV death, hHF or urgent visit due to HF in patients with type 2 diabetes and chronic kidney disease (SCORED), or after a recent episode of decompensated HF (SOLOIST-WHF). The studies were terminated early due to loss of funding. Higher incidence of diarrhea was observed, as well as genital mycotic infections, volume depletion, and diabetic ketoacidosis (SCORED) or hypoglycemic episodes (SOLOIST-WHF)	SCORED [91] SOLOIST-WHF [92]
GLP-1 receptor agonist	Albiglutide	30, 50 mg QW SC	Proven reduction in 3-point MACE and CV death in population of patients with risk factors for/or previous ASCVD. Currently not marketed	HARMONY Outcomes [15]
	Dulaglutide	0·75, 1·5 mg QW SC	Proven reduction in 3-point MACE in population of patients with risk factors for/or previous ASCVD. Improvement in secondary renal composite outcome	REWIND [17, 93]
	Exenatide XR	2 mg QW SC	Proven CV safety in population of patients with risk factors for/ or previous ASCVD	EXSCEL [14]
	Liraglutide	1·2–1·8 QD SC	Proven reduction in 3-point MACE and CV death in population of patients with risk factors (minority) or with previous (majority) ASCVD	LEADER [11]
	Lixisenatide	10, 20 mcg QD SC	Proven CV safety in population of patients with recent acute coronary syndrome (ACS)	ELIXA [9]
	Semaglutide	0·5, 1 mg QW SC	A pre-approval trial demonstrated reduction in 3-point MACE and CVA, but showed an increased risk for worsening of retinopathy in population with mostly ASCVD or CKD but some patients with only CV risk factors. More effective than other GLP-1 RAs in weight reduction and glucose control	SUSTAIN-6 [12]
	Semaglutide (Oral)	7, 14 mg QD PO	In a smaller pre-approval trial, proven CV safety in population of patients with risk factors for/or previous ASCVD. Demonstrated reduction in CV death and all-cause mortality. A larger CVOT is ongoing	PIONEER-6 [54] SOUL [94]
DPP4- inhibitors	Alogliptin	12·5, 25 mg QD	Proven CV safety in population of patients with recent ACS	EXAMINE [83]
	Linagliptin	5 mg QD	Proven CV safety in population of patients with risk factors for/or previous ASCVD. No further CV benefit over sulfonylurea (SU)	CARMELINA [55] CAROLINA [63]
	Saxagliptin	2·5, 5 mg QD	Proven CV safety in population of patients with risk factors for/or previous ASCVD. Increased risk for hHF	SAVOR-TIMI 53 [84]
	Sitagliptin	25,50,100 mg QD	Proven CV safety in population of patients with previous ASCVD	TECOS [85]
	Vildagliptin	50 mg BID	CV safety not tested	
TZDs	Pioglitazone	15, 30, 45 mg QD	Proven CV safety and possibly efficacy, reduced 3-point MACE in patients with insulin resistance but without diabetes after previous cerebrovascular event. Increased risk of HF, weight gain, fractures in post-menopausal women	PROactive [95] IRIS [86]
Insulin	Degludec		Proven CV safety in population of patients with risk factors for/or previous ASCVD	DEVOTE [96]
	Glargine		Proven CV safety in population of patients with risk factors for/or previous ASCVD, with diabetes or pre-diabetes	ORIGIN [97]
Biguanides	Metformin		Proven CV efficacy in small population of patients with relatively new onset type 2 diabetes. In a subgroup of patients treated with sulfonylurea, added metformin was associated with increased diabetes related death	UKPDS [41, 42]

*ASCVD* atherosclerotic cardiovascular disease, *CKD* chronic kidney disease, *CROT* cardiorenal outcome trial, *CV* cardiovascular, *CVA* cerebrovascular accident, *CVD* cardiovascular disease, *CVOT* cardiovascular outcome trial, *DPP4i* dipeptidyl peptidase 4 inhibitors, *GLP-1* glucagon-like peptide-1, *HFrEF* heart failure with reduced ejection fraction, *hHF* hospitalization for heart failure, *MACE* major adverse cardiovascular events, *SGLT1* sodium glucose co-transporter type 1, *SGLT2* sodium glucose co-transporter type 2, *TZDs* Thiazolidinediones

### Primary care team

We suggest the following set of SIMPLE concepts, in order to make guidelines more useful for primary care teams, which treat most patients with type 2 diabetes (Box 2):
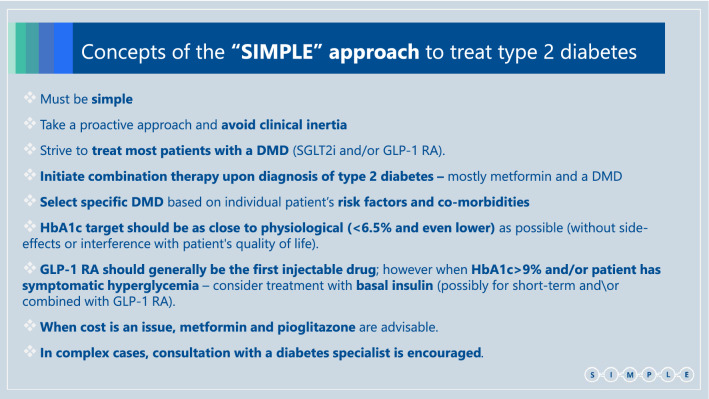

1. The guidelines must be **simple** enough - so they can be easily applied.
2. **Take a proactive approach** and intensify treatment to meet patients’ outcomes: **avoid clinical inertia**.
3. We should **strive to treat as many patients** as possible (and not only those with previous ASCVD/HF/CKD) **with** **diabetes/disease modifying drugs (DMDs)**; i.e. **SGLT2i and/or GLP-1 RA** that demonstrate the best evidence of improving patients’ prognosis.
4. Initial **combination therapy with metformin and a DMD** (either SGLT2i or GLP-1 RA) (or DPP4i, if a DMD is not possible) should be considered** for any patient upon diagnosis** of type 2 diabetes**. **
5. Specific **DMD selection** should rely on individual patient’s **risk factors and co-morbidities **and existing evidence.
6. Since DMDs do not increase the risk of hypoglycemia, aim for **HbA1c target that is as close to physiological** **(<6·5% and even lower)** as can be achieved without side-effects or decline in patient’s quality of life.
7. **GLP-1 RA should generally be the first injectable drug**; however when **HbA1c>9% and/or the patient has symptomatic hyperglycemia** - treatment with **basal insulin** (possibly for a short-term and preferably combination of basal insulin and GLP-1 RA, either as free or fixed ratio combination [FRC]) should be considered.
8. **When cost is an issue, metformin and pioglitazone can be considered**. However precautions should be taken when prescribing pioglitazone in patients who have a higher risk to develop HF or fractures. Use of sulfonylureas should be limited due to the relative high risk for side effects—hypoglycemia and weight gain (importantly, there is no clear evidence that they increase CV risk (55)).
9. In **complex cases** or in patients with severe presentation, consider other forms of diabetes or concomitance with precipitating conditions. In these cases **consultation with a diabetes specialist is encouraged**.

This short paradigm aims to empower the primary physicians, as they treat the majority of patients with type 2 diabetes. Nonetheless, we cannot over emphasize the importance of a multidisciplinary team in the process. This team includes nurse-practitioners, nurses, dieticians, diabetes educators, social workers, and coaches. Diabetologists/endocrinologists and other specialists should also be involved according to specific patient’s needs. All team members should cooperate to gain patient’s trust, collaboration and compliance. Coexisting cardiorenal risk factors such as hypertension, hyperlipidemia, obesity and liver function should be well corrected, if possible; it is worth noting that SGLT2i and GLP-1 RAs exert positive effects on many of them [20–22]. Chiefly, the team should encourage patients to follow a healthy lifestyle as a foundation for every treatment regimen.

### Life changing/Prevention

In 2015 we were all impressed by the first report that empagliflozin, an SGLT2i, may reduce cardiovascular (CV) and renal adverse outcomes [10, 56]. Presently, we have strong evidence that DMDs (SGLT2i and GLP1 RA) are prognosis changing [9–17] in the sense that they can reduce diabetes related complications far beyond their glucose lowering effect. Furthermore, these benefits are well supported by analyses of real-life data [57–61], including an analysis that compared different add-on GLA therapies in metformin-treated patients with moderate CV risk [62]. Thus, “life/prognosis changing” DMDs should become the cornerstone in the treatment of any patients with type 2 diabetes (Figs. 1, 2).

We acknowledge that this approach has some limitations. Many practitioners find sulfonylureas safe and effective for specific patients, as supported by the recent CAROLINA study [63] where the incidence of MACE was similar between linagliptin (a DPP4i) and glimepiride (a sulfonylurea). In addition, linagliptin did not reduce MACE compared with placebo in the accompanying CARMELINA trial [55]. However, the occurrence of moderate-severe hypoglycemia at one year was 20% in the glimepiride group, compared with less than 5% in the linagliptin group. Moreover, a recent registry-based analysis showed that initiation of treatment with DMDs was associated with lower rates of adverse renal (and CV [58]) outcomes compared with DPP4i and even more when compared with sulfonylureas [61]. We therefore feel that the use of sulfonylureas should be limited.

We recognize the financial burden caused by the use of costly DMDs in wider populations, both on the patients’ level and across all health systems and payers. This issue is specifically relevant in parts of the world that pressure on primary physicians to use less expensive drugs is greater. However, a large part of diabetes-associated expenditure is traced back to the cost of treating its complications [64, 65] which we hope to avoid or reduce with the use of these prognosis-changing agents. Cumulating data now support the cost-effectiveness of DMD use in different populations that participated in the CVOTs [66, 67]. Of course, this notion should also be systematically investigated in cost-effectiveness analyses in a manner that is independent of interest groups. In addition, the patents for DMDs will eventually expire, for some of them sooner than the others, for example a generic version of liraglutide may become available in 2023. Besides its practical use, we hope that this approach, together with other guidelines, will assist in leading the medical community as well as policy makers towards better use of DMDs.

Treatment of patients with eGFR < 60 ml/min/1.73 m^2^ requires further considerations. The lower glomerular filtration interferes with the glucose lowering effect of SGLT2i, yet their effect in improving hypertension, BMI and, most importantly, cardiorenal outcomes are still remarkable [68–70]. GLP-1 RAs were shown to be both safe and effective on improving glycemic control and BMI in several RCTs recruiting patients with eGFR < 60 ml/min/1.73 m^2^ [71–74].

Clinical judgement is necessary before prescribing DMDs. Caution is advised in SGLT2i treatment to those with high risk for diabetic ketoacidosis (DKA) or genital tract infections (GTIs). GLP-1 RAs should be avoided in patients with familial history of multiple endocrine neoplasia type 2 (MEN2) or medullary thyroid carcinoma, and precaution should be taken in patients that had idiopathic pancreatitis. We should also keep in mind that these are relatively new drugs, with little information regarding long term (> 5 years) safety. This paucity of data is partially compensated by the large number of participants in RCTs so far, allowing the detection of safety signals (e.g. increased risk for amputations in the CANVAS program [13], that have not been repeated in other trials). Registries should be constantly monitored for possibilities of yet unknown adverse effects. Yet, the current evidence is clear that the benefits of DMDs outweigh the risks.

As is generally accepted, the higher the background risk the higher the absolute risk reduction. Consequently, most CVOTs attempting to achieve statistically significant effects included patients with high CV- and renal risk, limiting our ability to extrapolate the results to lower risk populations. However, some more recent trials included patient populations without previous ASCVD, such as the DECLARE-TIMI 58 (reduction in hospitalization for HF with SGLT2i [16]) and REWIND (MACE reduction with GLP-1 RA [17]) trials, indicating a beneficial preventive effect for DMDs even in earlier stages of type 2 diabetes. Consequently, dulaglutide was the first type 2 diabetes medicine to receive FDA approval for the primary prevention of CVD in patients with type 2 diabetes [75]. Furthermore, recent registry-based analyses indicate that initiation of DMDs is associated with better renal outcomes across all tested subgroups, including patients with normal kidney function at baseline [59–61].

A recent analysis suggested that about a third of patients with type 2 diabetes are also diagnosed with CVD [76]. However, the true prevalence might be even higher and is related to differences in screening strategies across health systems. The global burden of diabetes-related end stage kidney disease (ESKD) has increased in the past decades, and other diabetes related complications affect millions worldwide [77]. Applying DMDs only to patients with established risk factors will undoubtedly miss many that evidently benefit from these drugs i.e. those that their high risk status was overlooked due to incomplete detection rate. All in all, the place and mission of DMDs in type 2 diabetes should be expanded from treatment of hyperglycemia to prevention of cardiovascular and kidney disease.

### (re)-Evaluation

Re-evaluation of patient’s therapy and treatment targets are needed on a regular basis to avoid clinical inertia [25, 78], and treatment approaches should provide the primary care team with easy tools. The decision to initiate injective therapy is an example of a situation that can make the primary care team confused or hesitant: a simple workflow can assist in the process.

**Fig. 3:**
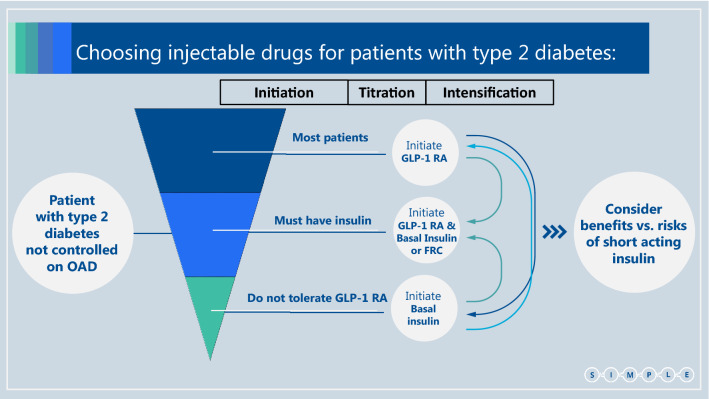
Choosing injectable drugs for patients with type 2 diabetes. *OAD* Oral antidiabetic drug, *GLP-1 RA* Glucagon-like peptide-1 receptor agonist

In current practice most patients use basal insulin as their first injectable drug, with only a small proportion starting with a GLP-1 RA. We concur with the ADA/EASD guidelines, suggesting flipping the pyramid upside down: most patients should commence with a GLP-1 RA injection and only a minority should start with basal insulin as the first injectable (including patients with symptomatic hyperglycemia and/or HbA1c > 9.0%, as above*)*. When the "first injection" does not suffice to control plasma glucose levels, treatment should be titrated and if needed intensified. This can be done either by the addition of insulin (or by transferring to fixed ratio combination—FRC) in patients treated with GLP-1 RA, or by the addition of GLP-1 RA (or transferring to FRC) when a patient is treated with basal insulin (Fig. 3).

Importantly, most of those requiring insulin should be considered for treatment with a combination of GLP-1 RAs—either as free combination or as FRC. Such a combination of injectable drugs has various advantages: it provides better glucose normalization with less hypoglycemic events compared with basal insulin and does not cause similar weight gain to the extent observed with basal insulin [79, 80]. It is also well tolerated and is associated with a lower rate of gastrointestinal side effects due to the slow titration, compared with GLP-1 RA alone [80]. However, since patients treated with insulin/GLP-1 RA combination tend have increased risk for hypoglycemia and increased weight gain than with GLP-1 RA alone, we do not recommend this combination as the first injectable but rather GLP-1 RA for most patients.

Due to the wide range of better options, short acting insulin therapy is moved down to the end of the list. Its usage in patients with type 2 diabetes should be carefully evaluated considering its advantages in glucose control compared with its side effects including high risk of hypoglycemia, weight gain and lower quality of life [81].

Lastly, management of patients with difficult to control diabetes requires special attention. Type 2 diabetes is still too often considered a condition resulting from poor adherence to lifestyle rather than a disease per se. Thus, consulting with a diabetes specialist is encouraged in patients that do not achieve glycemic control or in those with unique or alarming clinical features.

## Conclusions

Since the busy primary care teams treat most patients with type 2 diabetes world-wide, treatment approaches should be as simple and convenient as possible. Yet, the recent increase in good therapy options, the surge of relevant data, and the need to "personalize" treatment—including their risk factors and possible cardiorenal complications—make this mission more complex than ever before. The development and characterization of the Diabetes\Disease Modifying Drugs provide us with a golden opportunity to simplify the treatment of type 2 diabetes, while reducing the disease's complications. Early combination therapy with two and sometimes three of the DMDs (SGLT2i and GLP-1 RA) and metformin, and lower HbA1c targets (< 6.5%), may halt and even regress the pathological basis of diabetes and improve patient’s prognosis.

## Data Availability

Data sharing not applicable to this article as no datasets were generated or analysed during the current study.

## Abbreviations

ASCVD: Atherosclerotic cardiovascular disease
CV: Cardiovascular
CVD: Cardiovascular disease
CVOTs: Cardiovascular outcomes trials
DKA: Diabetic ketoacidosis
DMDs: Diabetes/disease modifying drugs
DPP4i: Dipeptidyl peptidase 4 inhibitors
eGFR: Estimated glomerular filtration rate
ESKD: End stage kidney disease
FDA: Food and Drug Administration
FRC: Fixed ratio combination
GLAs: Glucose lowering agents
GLP-1 RA: Glucagon-like peptide-1 receptor agonists
GTI: Genital tract infection
HF: Heart failure
MACE: Major adverse cardiovascular event
MEN2: Multiple endocrine neoplasia type 2
RCT: Randomized controlled trial
SGLT2i: Sodium glucose co-transporter type 2 inhibitors
TZD: Thiazolidinedione
UACR: Urinary albumin to creatinine ratio
